# Emerging Portable Technology Provides Preliminary Predictive Models of Maximum Velocity Sprinting in National Australian Track Athletes: A Foundation for Measuring Sprint Performance in the Field

**DOI:** 10.3390/sports14080346

**Published:** 2026-08-11

**Authors:** Timothy Sayer, Nicholas Cross

**Affiliations:** 1 Department of Physiotherapy, La Trobe University, Melbourne, VIC 3086, Australia; 2 Department of Physiotherapy, The University of Melbourne, Melbourne, VIC 3010, Australia; 3 Melbourne CBD Physiotherapy and Sports Medicine Clinic, Ground Floor, 179 Queen Street, Melbourne, VIC 3000, Australia

**Keywords:** sprint performance, sprint biomechanics, maximal velocity, portable technology

## Abstract

Maximal velocity (MaxV) is a key determinant of sprint performance, yet its biomechanical predictors have rarely been examined in the field. This exploratory study developed preliminary predictive models of sprint time and MaxV in national level Australian track sprinters using portable technology. Twenty-eight sprinters (16 male, 12 female) each completed two maximal 60 m sprints recorded with a MuscleLab LaserSpeed radar, SportScientia Techlayer instrumented insoles, and VueMotion kinograms. The two trials per athlete were averaged (intraclass correlation 0.83–0.98) to give 28 independent observations, and best-subsets multiple regression selected by leave-one-out cross-validated R^2^ subject to all variance inflation factors below five was used to model 60 m and 20 m sprint time and MaxV. The 60 m sprint time was almost entirely explained by MaxV alone (R^2^ = 0.94, cross-validated R^2^ = 0.93), an expected mechanical relationship. The 60 m MaxV was associated with normalised peak force, vertical loading rate, time to peak force and thigh angular velocity (R^2^ = 0.85, cross-validated R^2^ = 0.78). The 20 m sprint time was predicted by 20 m MaxV (R^2^ = 0.80), while vertical loading rate was the only significant predictor of 20 m maximal velocity (β = 0.65, R^2^ = 0.59). These preliminary findings suggest that portable instrumented insoles, sagittal plane kinematics and infrared radar may assist in identifying candidate biomechanical variables associated with MaxV in the field. Given the small sample and absence of external validation, the models require confirmation in larger, independent cohorts.

## 1. Introduction

Sprint performance is critical for success in both track and field sprint events and various field-based sports. Sprinting is a highly complex motor task influenced by a range of neuromuscular, biomechanical, and anatomical factors [1,2]. Understanding which factors are critical to sprint performance has long been desired by coaches, sports scientists and health professionals, as key predictors of sprint performance could lay the foundation for tracking athletic progress of targeted interventions. Moreover, the identification of key sprint predictors may provide insight for methods to reduce soft tissue injuries such as hamstring tears, given that many of these injuries occur during maximal sprint efforts [3].

It is widely accepted that a key determinant of sprint performance is the ability to produce and sustain higher maximal velocity (MaxV) through the production of increased horizontal and vertical ground reaction forces and optimised sagittal plane running kinematics [1,2]. Previous research by Morin and colleagues [1] recruited 12 sub-elite sprinters and examined maximal sprinting on an instrumented treadmill compared to a 100 m sprint time and found that the ratio of force and net horizontal ground reaction force were strongly correlated (R^2^ = 0.64) to maximal velocity. Furthermore, evidence from Cross et al.’s [4] systematic review highlights that peak horizontal power has also been shown to strongly correlate with sprint time, and kinematic variables such as step length, step frequency, and contact time also demonstrate predictive value, although with slightly lower R^2^ values (0.54–058). From a kinematic perspective, higher thigh angular velocity through the swing phase, reduced knee joint excursion during the stance phase and reduced thigh separation between touch down limb and contralateral swing limb were noted [5,6,7]. Consequently, these studies highlight that sprint performance is strongly correlated with biomechanical variables associated with foot–ground interaction and the application of force quickly to achieve a higher MaxV.

Despite the well-founded studies on sprint biomechanics and the important role of MaxV on sprint performance, the aforementioned studies are primarily conducted in laboratory settings that are somewhat unnatural to the athlete’s environment. Furthermore, the expansion of portable technology, such as wireless insoles with integrated inertial measurement units (IMU) and force sensors, high-frequency radar and lower body kinograms via mobile video footage now enable real-time, in-field assessment of sprint biomechanics in an athletes familiar training and competitive environment and represents a novel way of monitoring athletic development and potentially injury risk. Furthermore, as MaxV, rather than sprint time, represents the trainable mechanical quality that coaches seek to develop and that transfers most readily across sprint events, MaxV was treated as the primary outcome of interest in the present study. Consistent with the force velocity theory [1,2] and the minimum-requirements framework of Clark [8], we hypothesised a priori that variables reflecting rapid, high-magnitude vertical force application, would be the strongest predictors of MaxV. Hence, the primary aim of this study was to characterise and develop preliminary predictors of sprinting performance using portable technology among national level Australian track and field sprinters.

## 2. Materials and Methods

### 2.1. Study Design

This was a cross-sectional observational study designed to identify biomechanical predictors of maximal sprint performance in elite track and field athletes. A field-based assessment was conducted using wearable sensor technology, laser timing, and motion analysis tools across standardised sprint trials. Twenty-eight elite Australian track national-level competitors and Olympic qualifier sprinters (mixed sample: 16 male, 12 female, 69.21 ± 8.45 kg, 1.74 ± 0.08 m) participated in this study. Inclusion criteria were Australian national level competition status competing in 100, 200 or 400 m sprints and no lower-limb injury within 6 months prior to testing. All data were collected in a single testing session in December 2024. All participants provided informed consent in accordance with ethics approval of the University of Melbourne by the human ethics research committee (ethics ID 30942).

#### Sprint Protocol

**Figure 1:**
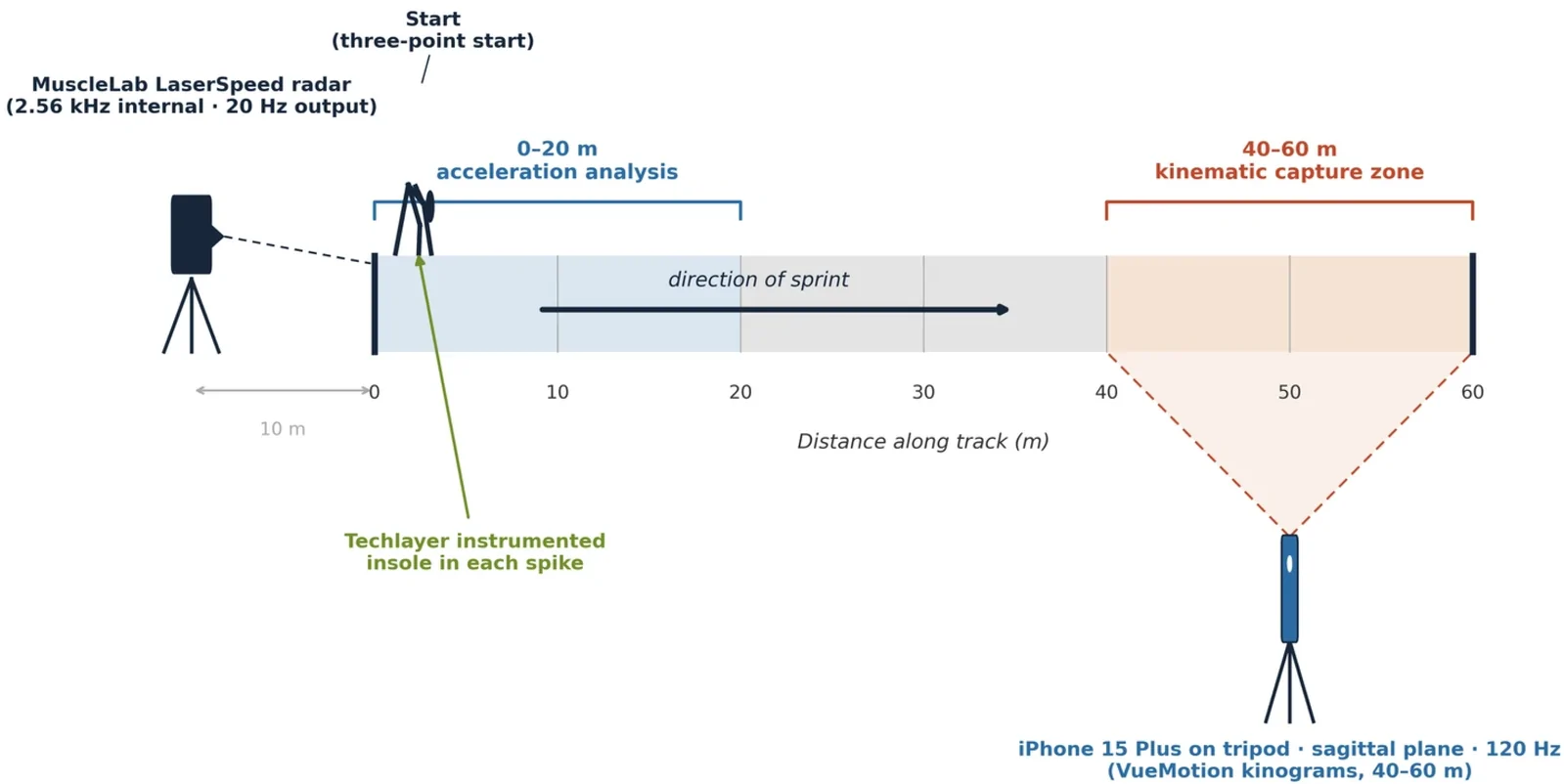
Schematic diagram of test protocol setup for capturing data using MuscleLab Laser Speed, SportScientia Techlayer and Vuemotion.

Athletes completed their regular warm-up routines as per their individual training practices. Each athlete then performed two maximal 60 m sprints on a Mondo athletics track, starting from a standardised three-point position (Figure 1). Instructions were given to the athlete to begin on the start line and from a three-point stating position to begin in their own time. This was chosen to minimise the influence of reaction time on sprint performance. Adequate rest (>10 min) was provided between sprint efforts to minimise fatigue influencing their maximal sprint efforts.

### 2.2. Timing and Maximal Velocity

Sprint time and maximal velocity across 60 m and 20 m were measured using a MuscleLab LaserSpeed system (Ergotest, Porsgrunn, Norway) with established validity and reliability measuring sprint performance [9]. The radar was positioned 10 m behind the starting line and sampling internally at 2.56 kHz with a 20 Hz output. The radar was set up with the use of MuscleLab software (V10.241.117.5374, February 2025) and set to auto-detect mode for each sprint whereby recording would begin once the athlete’s centre of mass was moving away from the radar at >0.01 m/s. All athletes wore a reflective vest to ensure accurate data capture. The device recorded time in seconds (s) to 60 m and segmented data into intervals of 10 m. For the 60 m data, the maximal velocity across 60 m (MaxV, m/s) and maximal step length x step frequency (MaxStepFreq, m•Hz) was extracted. For the 20 m acceleration, the 20 m maximal velocity (MaxV_20m), percentage of maximal velocity at 20 m relative to 60 m (percent_maxV60) was extracted for each sprint from the MuscleLab software using their custom algorithms. Data was extracted by Microsoft Excel CSV files for each athlete.

### 2.3. Spatiotemporal and Force-Time Variables

**Figure 2:**
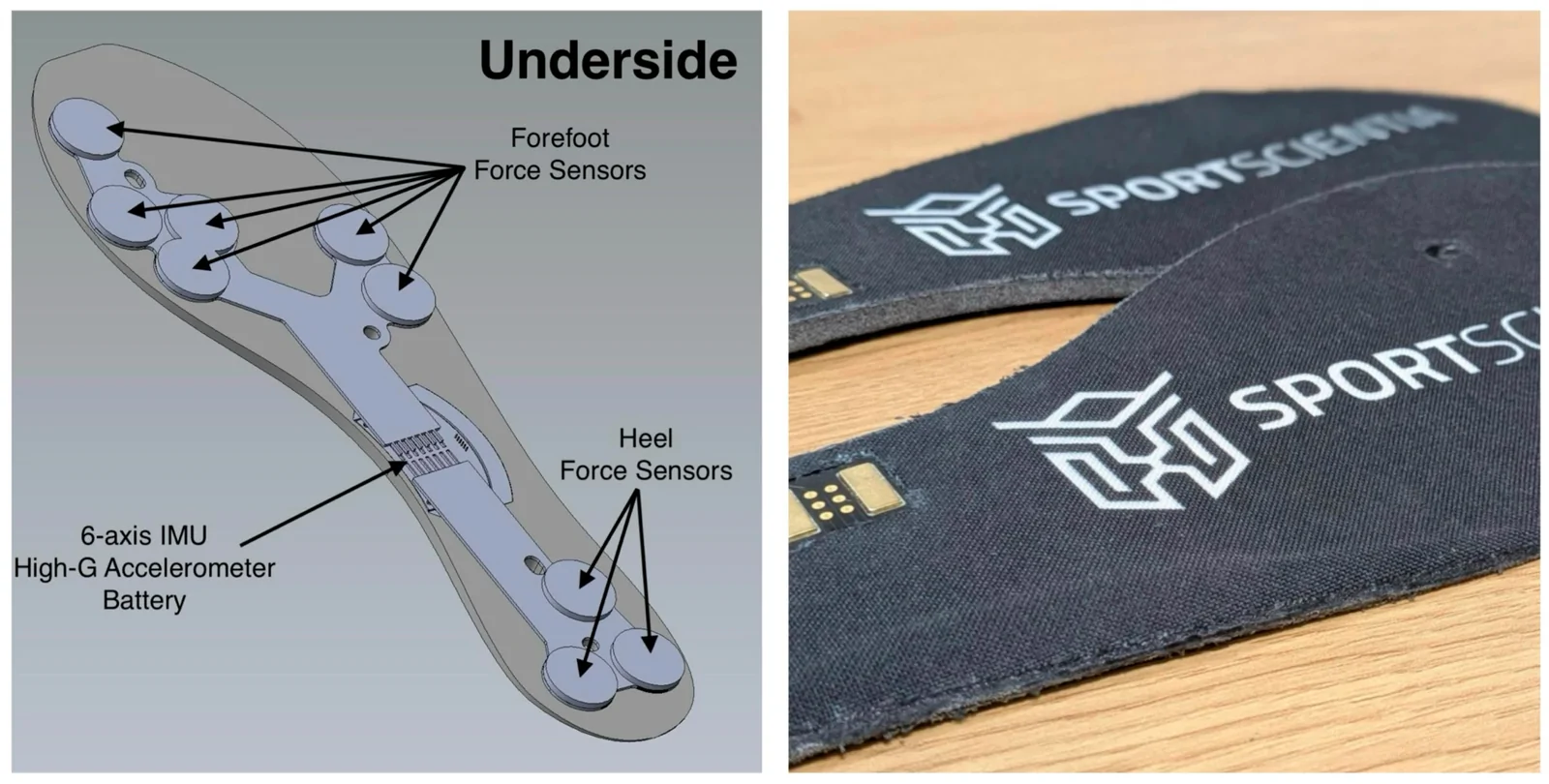
Schematic illustration of the SportScientia Techlayer. A thin (1–5 mm) and lightweight (45 g) insole integrates a 6-axis Inertial Measurement Unit (IMU) comprising a 16G accelerometer (500 Hz), a 200G high-acceleration sensor (500 Hz), and a gyroscope (2000 dps at 500 Hz).

A wireless Techlayer insole (SportScientia PTE. LTD Incorporated, Singapore) was inserted into each athlete’s sprint spike before the sprint. This thin (1–5 mm) and lightweight (45 g) insole integrates a 6-axis Inertial Measurement Unit (IMU) comprising a 16G accelerometer (500 Hz), a 200G high-acceleration sensor (500 Hz), and a gyroscope (2000 dps at 500 Hz). In addition, the Techlayer insole has nine force sensors throughout the forefoot and heel of the insole (Figure 2). Previous research has demonstrated that instrumented insoles devices utilising IMU’s and accelerometers have appropriate gait related reliability and validity [10,11].

Before each session, the Techlayer was charged using a SportScientia charging box that mounts to the battery in the middle of the insole where the IMU is located. Once charged, the Techlayer insole was paired and synced with the SportScientia software (Desktop App Version 0.0) on a laptop at the track and placed inside the spike of each athlete. The weight of the athlete walking automatically cued the recording of data from each insole (left and right side) through the detection of movement and pressure on the force sensors and IMU. Following each sprint, the Techlayer was removed from the spike and mounted to the charging box and connected to the internet whereby data was uploaded to the SportScientia cloud data hub using their patented software. Each sprint was identified using a custom SportScientia algorithm that identified peak force (N), peak force **÷** contact time (peakforce_CT, N/ms) contact time (CT, ms), impulse (kN/s), vertical loading rate (VLR, N/s), peak speed (m/s), time between ipsilateral initial contact to intial contact (IC, ms), minimum acceleration magnitude (min_accel_magnitude, G), time to peak force (ms), and swing speed (m/s) throughout the sprint. Every step across the 60 m sprint was collated and averaged for each variable across 0–60 m and 0–20 m for analysis and exported to a Microsoft Excel CSV file.

### 2.4. Kinematics

Kinematics were collected using VueMotion Labs Pty Ltd., (Sydney, Australia) kinograms over the final 20 m of each sprint using an iPhone 15 Plus (Apple Incorporated, Cupertino, CA, USA) mounted to a tripod capturing the sagittal plane running of each athlete at 120 Hz. The video footage of each sprint from 40 to 60 m was downloaded from the iPhone library and uploaded to the VueMotion software (version 1.2) and analysed using its proprietary algorithm, whereby a calibration method is used to accurately map out ground-based measures. The knee angle at full support in the stance phase (knee_angle_full_support, °), thigh angular velocity (TAV, rad/s), thigh split angle at initial contact (IC, °) and positive running score (half angle of the thigh separation at toe off to the vertical line from the hip) were extracted and reported as means for the 60 m sprint within the 40–60 m capture zone. VueMotion data was not utilised for the 0–20 m analysis as there was no data capture in this phase of the sprint. Markerless, smartphone-based motion capture has emerged as a valid and reliable means of quantifying lower-limb kinematics in the field [12,13]. Platforms that reconstruct three-dimensional kinematics from smartphone video, such as OpenCap, have demonstrated acceptable concurrent validity against marker-based systems and good between-session reliability for lower-limb joint angles, albeit with joint- and task-specific accuracy [12,14,15].

### 2.5. Statistical Analysis

Descriptive statistics (mean ± SD) were calculated for all variables across both the 0–60 m and 0–20 m phases (Table 1). Because each athlete performed two maximal sprints, the two trials were first averaged to a single athlete-level value for every variable, yielding 28 independent observations and avoiding the non-independence (pseudoreplication) that would arise from treating the 56 sprints as independent. Trial-to-trial reliability was quantified with the intraclass correlation coefficient (ICC, two-way, single measures) and was good-to-excellent for the outcome variables (60 m time ICC = 0.94, MaxV = 0.98; 20 m time ICC = 0.83, MaxV_20m = 0.97), supporting aggregation. Twenty-one candidate variables were available. For each dependent variable (60 and 20 m sprint time and maximal velocity), an exhaustive best-subsets procedure enumerated all linear models containing one to five predictors. Variables that were mathematically dependent on the outcome, for example, maximum step length × frequency and peak speed, which are algebraically equivalent to velocity, were excluded from the corresponding MaxV models, and near-redundant predictors (for example, absolute versus normalised peak force) were governed by a mutual-exclusion constraint permitting at most one per model. To control multicollinearity, only models in which all predictors had a variance inflation factor (VIF) below five were retained. The final model for each outcome was the retained subset that maximised the leave-one-out cross-validated coefficient of determination (predicted R^2^, computed analytically via the PRESS statistic), this criterion favours parsimony and guards against overfitting. Selected models were fitted by ordinary least squares in SPSS v27.0 (IBM Corp, Armonk, NY, USA). For each model, we report unstandardised (B) and standardized (β) coefficients with 95% confidence intervals, *p*-values and VIF, together with in-sample R^2^, adjusted R^2^, SEE, and the cross-validated (PRESS) R^2^ and its difference from the in-sample R^2^ (optimism) as an index of overfitting. Residual diagnostics (Shapiro–Wilk normality, Breusch–Pagan homoscedasticity, Cook’s distance and leverage) were examined for each final model. Given the modest sample, the maximum model size of five predictors corresponded to an events-per-variable ratio as low as 5.6; the models were therefore treated as exploratory and no *a priori* power calculation was performed. Reporting was informed by the STROBE guideline for cross-sectional studies and, given the predictive aim, by the relevant reporting principles of the TRIPOD statement. Significance was set at *p* < 0.05.

## 3. Results

### 3.1. 60 m Sprint Analysis

**Figure 3:**
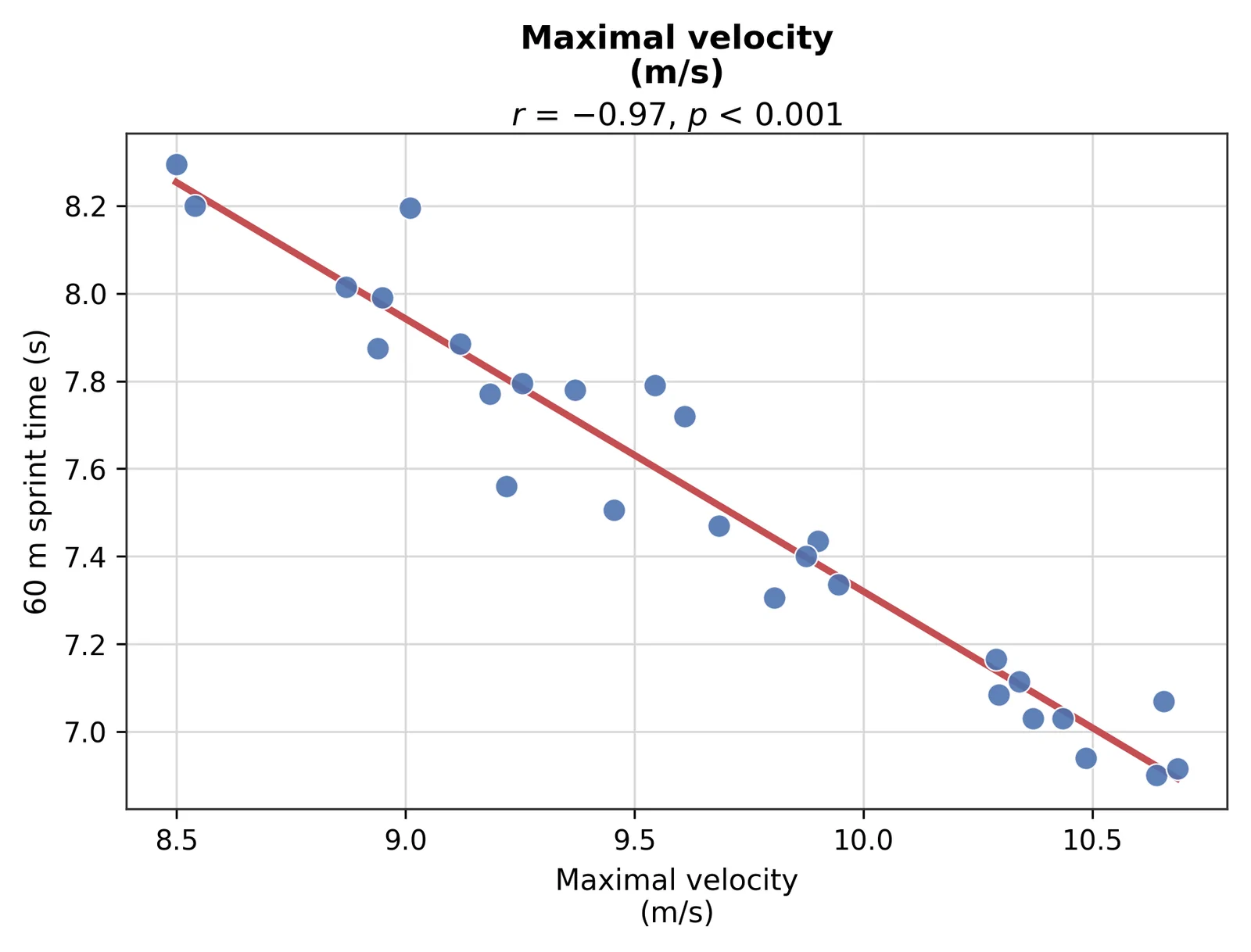
Relationship between 60 m sprint time and its single retained predictor, maximal velocity (MaxV). The scatterplot shows 60 m sprint time against MaxV with the ordinary least-squares line of best fit, the bivariate Pearson correlation (r) and the predictor’s *p* value within the model.

All regression models are summarised statistically in Tables S1–S4 of the Supplementary Materials. Following aggregation of the two sprints per athlete to athlete-level means (n = 28), the model for 60 m sprint time retained MaxV as the only predictor to survive cross-validated selection under the variance inflation factor (VIF) constraint. This single-predictor model was statistically significant, F(1, 26) = 406.17, *p* < 0.001, and accounted for 94.0% of the variance in sprint time (R^2^ = 0.940, adjusted R^2^ = 0.938, SEE = 0.107; leave-one-out cross-validated R^2^ = 0.932, optimism = 0.008). MaxV was a very strong negative predictor (B = −0.62, β = −0.97, *p* < 0.001, 95% CI [−0.686, −0.559]), indicating that higher MaxV was strongly associated with shorter sprint times (Figure 3). No additional biomechanical variable improved cross-validated prediction, consistent with 60 m sprint time being mechanically dominated by, and largely embedded within MaxV. At the bivariate level, 60 m sprint time was very strongly and negatively correlated with maximal velocity (r = −0.97, *p* < 0.001; Figure 3).

**Figure 4:**
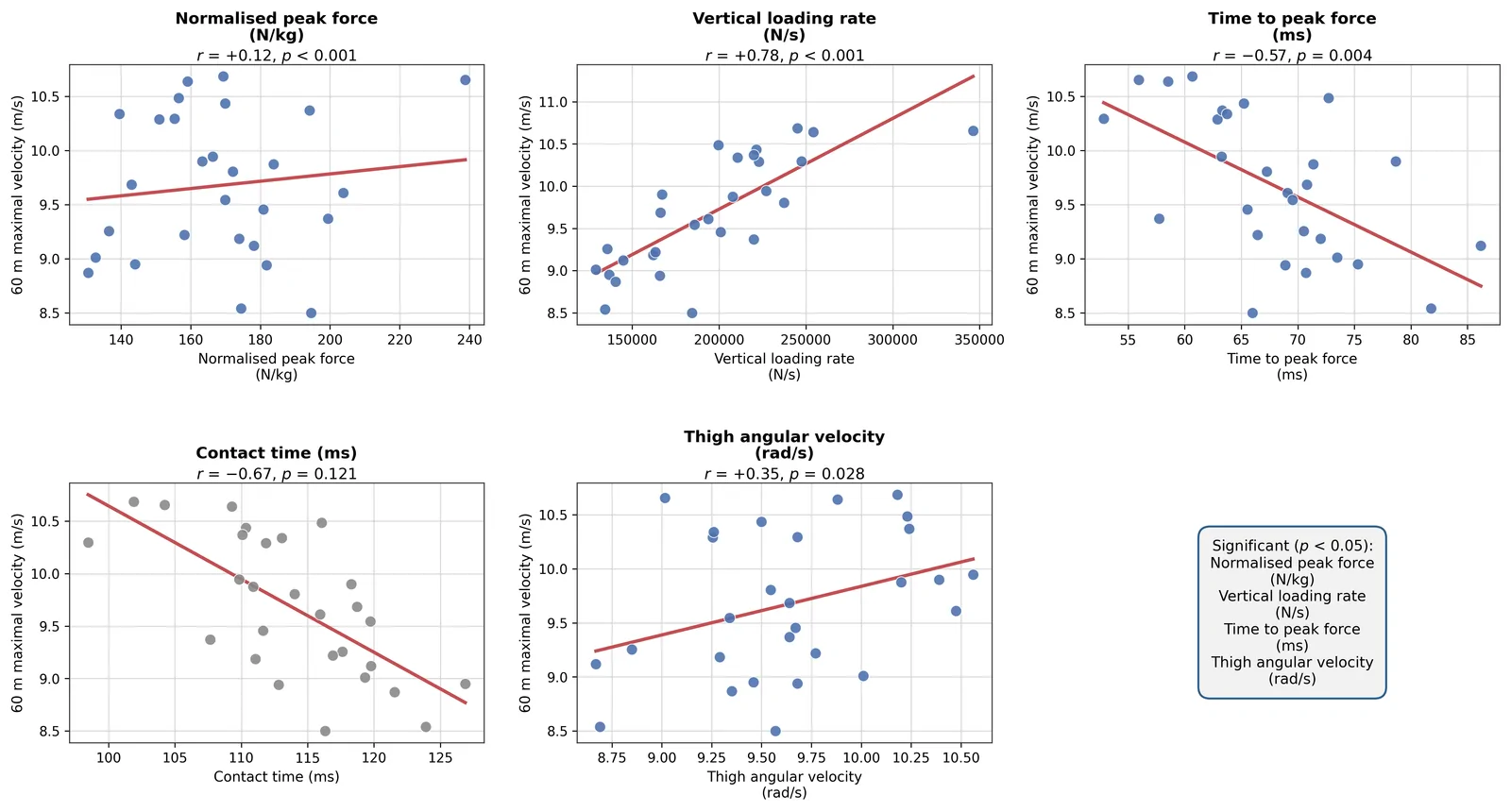
Best-subset multiple linear regression model for 60 m MaxV. Each panel shows MaxV against one of the five model predictors (normalised peak force, vertical loading rate, time to peak force, contact time, and thigh angular velocity), with the line of best fit, the bivariate Pearson correlation (r) and the predictor’s *p* value within the model. Significant predictors (*p* < 0.05) are plotted in blue and non-significant predictors in grey.

The model for 60 m MaxV retained five predictors under cross-validated selection with all VIF < 5, and was statistically significant, F(5, 22) = 25.00, *p* < 0.001, explaining 85.0% of the variance in MaxV (R^2^ = 0.850, adjusted R^2^ = 0.816, SEE = 0.285; cross-validated R^2^ = 0.775, optimism = 0.075; events-per-variable ≈ 5.6). Vertical loading rate (β = 1.24, *p* < 0.001), normalised peak force (β = −0.55, *p* < 0.001), time to peak force (β = 0.52, *p* = 0.004) and thigh angular velocity (β = 0.20, *p* = 0.028) were significant independent contributors, whereas contact time did not reach significance (β = −0.26, *p* = 0.121; Figure 4, Table S2 Supplementary File). The standardised coefficients for normalised peak force and time to peak force were opposite in sign to their bivariate correlations with MaxV (r = 0.12 and −0.57, respectively), indicating suppressor effects. These predictors should therefore be interpreted, together with vertical loading rate which carried the highest variance inflation factor in this model (VIF = 4.48, approaching the a priori threshold of 5) as a correlated cluster of force–time variables contributing within the multivariable model rather than as independent mechanical determinants. Bivariate correlations between 60 m MaxV and each model predictor are displayed in Figure 4. MaxV was most strongly correlated with vertical loading rate (r = 0.78, *p* < 0.001).

### 3.2. 20 m Acceleration Analysis

**Figure 5:**
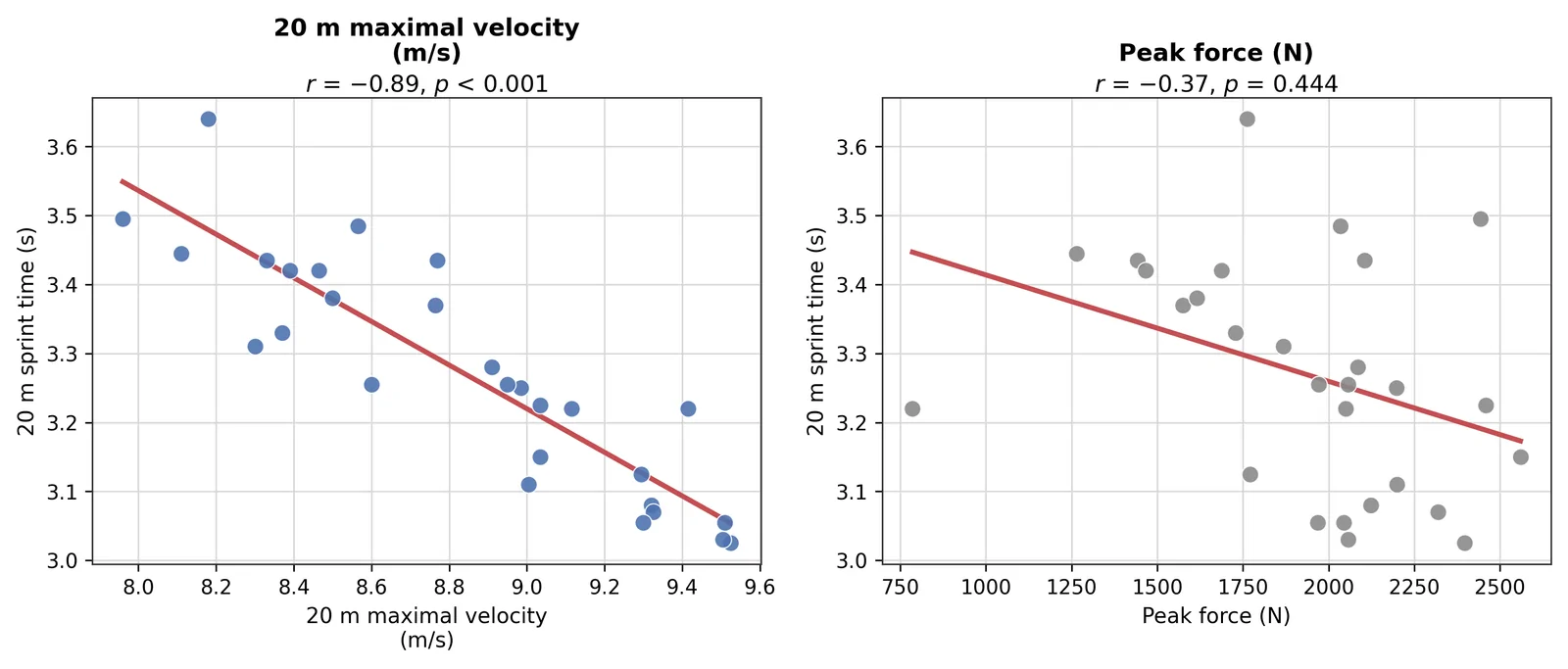
Model for 20 m sprint time. Each panel shows 20 m sprint time against a model predictor (20 m maximal velocity and peak force), with the line of best fit, the bivariate Pearson correlation (r) and the predictor’s *p* value within the model. Significant predictors (*p* < 0.05) are in blue and non-significant predictors in grey.

The model for 20 m sprint time was statistically significant, F(2, 25) = 49.07, *p* < 0.001, accounting for 79.7% of the variance (R^2^ = 0.797, adjusted R^2^ = 0.781, SEE = 0.078; cross-validated R^2^ = 0.758, optimism = 0.039). Twenty m maximal velocity (MaxV_20m) was the only significant predictor (B = −0.31, β = −0.87, *p* < 0.001, 95% CI [−0.377, −0.237]), while peak force did not reach significance (β = −0.07, *p* = 0.444; Figure 5, Table S3 Supplementary File), again reflecting the expected dominance of maximal velocity over sprint time. At the bivariate level, 20 m sprint time was strongly and negatively correlated with MaxV_20m (r = −0.89, *p* < 0.001; Figure 5).

**Figure 6:**
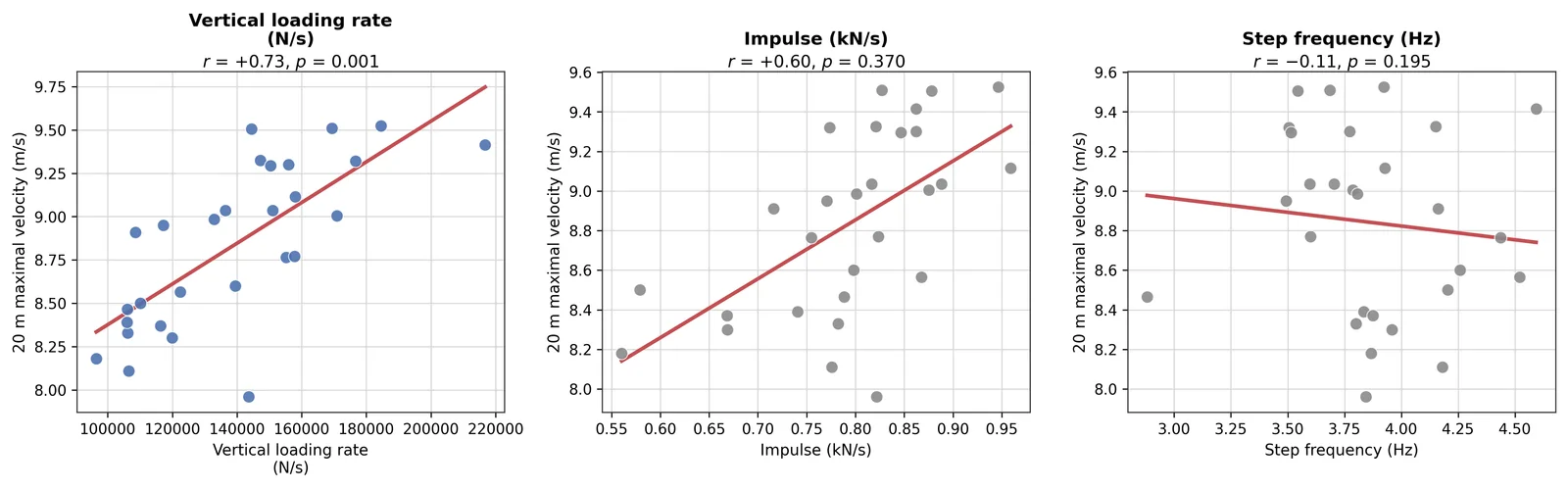
Model for 20 m maximal velocity (MaxV_20m). Each panel shows MaxV_20m against a model predictor (vertical loading rate, impulse and step frequency), with the line of best fit, the bivariate Pearson correlation (r) and the predictor’s *p* value within the model. Significant predictors (*p* < 0.05) are in blue and non-significant predictors in grey.

The model for MaxV_20m was statistically significant, F(3, 24) = 11.38, *p* < 0.001, explaining 58.7% of the variance (R^2^ = 0.587, adjusted R^2^ = 0.536, SEE = 0.319; cross-validated R^2^ = 0.496, optimism = 0.091). Vertical loading rate was the only significant predictor (B = 1.05 × 10^−5^, β = 0.65, *p* = 0.001, 95% CI [4.51 × 10^−6^, 1.64 × 10^−5^]), whereas impulse (β = 0.16, *p* = 0.370) and step frequency (β = −0.18, *p* = 0.195) did not reach significance (Figure 6, Table S4 Supplementary File). Finally, MaxV_20m was most strongly correlated with vertical loading rate (r = 0.73, *p* < 0.001), with weaker associations for impulse (r = 0.60) and step frequency (Figure 6).

## 4. Discussion

This study is the first to explore predictors of maximal velocity sprinting using portable technology in national level sprinters across 60 and 20 m. As expected, and in agreement with previous research [1,2], we found that MaxV was the most influential predictor of 60 m sprint performance, explaining over 93% of the variance. Moreover, the correlation between MaxV and time was also significantly high at 97% (r =−0.97). Our research extends previous findings by Healy et al. [16] whereby maximal velocity was correlated with 100 m sprint times (r = −0.97) across international events reconstructed from video footage to derive a time x velocity curve. Moreover, Tam and Yao [17] utilised machine learning models to analyse elite-level 100 m sprint data and reported a strong negative correlation between MaxV and overall sprint time, confirming that athletes who attain higher speed achieve quicker times. Importantly, these findings highlight the ability for portable technology to also identify the importance of MaxV, similar to previously published laboratory studies and thereby provides new methods for coaches, sport scientists and health professionals to measure this data in the field [18].

Beyond the dominance of MaxV over sprint time, the more informative models were those predicting MaxV itself. For 60 m MaxV, the mechanical qualities associated with sprinting explained 85.0% of the variance (cross-validated R^2^ = 0.78). After excluding variables that are algebraically equivalent to velocity (maximum step length × frequency and peak speed), 60 m maximal velocity was associated with vertical loading rate, normalised peak force, time to peak force and thigh angular velocity. The prominence of vertical loading rate and rapid force development is consistent with Morin et al. [1] and Clark [8], who identified the capacity to apply large vertical forces during progressively shorter ground contacts as a determinant feature of high-velocity sprinting, while the contribution of thigh angular velocity aligns with research identifying rapid swing dynamics as characteristic of maximal-velocity running [8,19]. Effective sprinters therefore appear to apply force rapidly and reposition the swing limb quickly to sustain forward propulsion [20,21]. However, because several of these variables are mechanically inter-related, and because normalised peak force and time to peak force displayed suppressor effects, these predictors should be interpreted as statistical contributors within the multivariable model rather than as independent mechanical determinants of MaxV.

Regarding the 20 m findings, MaxV_20m was again the strongest predictor of sprint time, with the model explaining 79.7% of the variance. Similar to the 60 m results, this highlights the importance of achieving high velocities rapidly during the acceleration phase [20], a phase characterised by progressively shifting kinetic demands across successive steps [22] and systematic changes in lower-limb kinematics as sprinters transition from acceleration to MaxV [23]. Peak force was retained in the 20 m time model but did not reach significance, consistent with the expected dominance of MaxV over sprint time. Nonetheless, the moderate bivariate association between force-related measures and performance supports the work of Morin et al. [20], who showed that acceleration capacity is associated with the ability to produce greater net horizontal impulse while minimising braking. In the corresponding model for MaxV_20m, vertical loading rate was the only significant predictor (bivariate r = 0.73), with weaker associations for impulse and step frequency. This model explained less variance (R^2^ = 0.587, cross-validated R^2^ = 0.50) than the 60 m model for MaxV, likely reflecting the absence of kinematic kinogram data over the shorter distance and the smaller number of retained predictors. Collectively, these findings indicate that vertical force application and loading rate are candidate correlates of early-phase MaxV that portable systems can capture, and might suggest that interventions targeting 20 m performance prioritise the development of MaxV through rapid force application into the ground.

While the findings of this study are novel there are some important noteworthy limitations. First, although the two sprints per athlete were averaged to respect the clustered data structure, the resulting sample (n = 28) remains modest relative to the number of candidate predictors, giving an events-per-variable ratio as low as 5.6, combined with best-subsets variable selection, the models retain a risk of overfitting, and the reported R^2^ values, though accompanied by only modest optimism may still be optimistic. Second, the models were developed and internally validated on a single dataset, with no external validation in an independent cohort, so the predictors identified should be regarded as candidate rather than definitive determinants. Third, several candidate variables are mathematically related, so individual coefficients, particularly where suppressor effects were observed should be interpreted as statistical contributions within the multivariable model rather than as independent mechanical mechanisms. Finally, the models combine measurements from three separate portable technologies, each with its own measurement error. The cumulative measurement uncertainty across radar, instrumented insoles and smartphone kinematics may attenuate or inflate individual associations and warrants dedicated validation. The pooled, mixed-sex composition of the cohort (16 male, 12 female) is a further consideration, although pooling was retained to preserve statistical power given the low events-per-variable ratio, it cannot rule out sex-specific differences in the identified predictors, and generalisation of the models across sexes should therefore be approached with caution. Moreover, because the same 28 observations were used both to search the large space of candidate models (one to five predictors drawn from 21 candidates) and to estimate the cross-validated R^2^, the reported optimism (in-sample minus PRESS R^2^) likely underestimates the predictive shrinkage that an independent sample would reveal. Hence, nested cross-validation or an external holdout sample would be required for an unbiased estimate of predictive performance in future work.

### Practical Applications

This study demonstrates that portable, field-based technologies, namely instrumented insoles and infrared radar, can generate preliminary predictive models of sprint performance in elite track athletes, offering practitioners a practical alternative to laboratory-based assessment. MaxV emerged as the strongest predictor of both 60 m and 20 m sprint time. As MaxV is mechanically embedded within sprint time this relationship is expected rather than novel, but it nonetheless reinforces the value of monitoring and developing top-end speed in this population. Among the mechanical qualities associated with MaxV, vertical loading rate, normalised peak force and time to peak force, together with thigh angular velocity over 60 m suggest that training strategies targeting the rapid application of large vertical forces and efficient swing-limb mechanics may be particularly relevant for enhancing sprint speed. Despite these findings, as the present models were derived from a small cohort of sprinters using variable-selection procedures and were not externally validated, the identified variables should be regarded as candidate rather than definitive determinants and as such practitioners should confirm these associations within their own athletes before applying them for performance prediction or talent identification.

## 5. Conclusions

This exploratory study demonstrated that portable, field-based technology can generate preliminary predictive models of sprint performance in national level sprint athletes. Over 60 m, sprint time was almost entirely explained by MaxV (R^2^ = 0.94), confirming the expected mechanical relationship, while MaxV itself was associated with normalised peak force, vertical loading rate, time to peak force and thigh angular velocity (R^2^ = 0.85). Across 20 m, sprint time was dominated by MaxV_20m, and vertical loading rate was the only significant predictor of 20 m maximal velocity (R^2^ = 0.59). These findings highlight vertical force application and loading rate as candidate correlates of maximal velocity that portable systems can capture in the field.

## Figures and Tables

**Table 1:** Descriptives summary of variables obtained from MuscleLab LaserSpeed, SportScientia Techlayer and VueMotion during 60 and 20 m sprints. Data is represented as mean ± standard deviation (SD).

Technology	Variables	0–60 m (Mean ± SD)	0–20 m (Mean ± SD)
MuscleLab Radar	Time (s)	7.52 ± 0.43	3.27 ± 0.17
	MaxV (m/s)	9.68 ± 0.66	-
	MaxV_20m (m/s)	-	8.84 ± 0.47
	MaxStepFreq (m•Hz)	9.37 ± 0.72	-
	Percentage of 60 m maximal velocity	100	91.46 ± 2.15
SportScientia Techlayer	Peak force (N)	1983.11 ± 408.65	1929.96 ± 403.67
	Normalised peak force (N/kg)	28.94 ± 6.31	28.12 ± 6.00
	Peak force_contact time (N/ms)	101.78 ± 20.00	73.86 ± 12.42
	min_accel_magnitude (G)	37.10 ± 10.17	24.24 ± 6.14
	Time to peak force (ms)	67.87 ± 7.68	81.81 ± 9.34
	CT (ms)	115.16 ± 9.20	132.35 ± 8.86
	Impulse (kN/s)	0.85 ± 0.09	0.80 ± 0.09
	VLR (N/s)	195,396.62 ± 48,156	139,512.62 ± 29,280.70
	Peak speed (m/s)	9.30 ± 0.67	9.30 ± 0.66
	Peak swing speed (m/s)	15.16 ± 1.08	14.57 ± 0.96
	Time between ipsilateral limb IC to IC (ms)	461.25 ± 16.41	457.83 ± 18.40
VueMotion Kinograms	knee_angle_full_support (°)	149.93 ± 6.00	-
	TAV (rad/s)	9.64 ± 0.58	-
	Thigh split angle at IC (°)	7.66 ± 15.32	-
	Positive running score	22.25 ± 4.38	-
	IC distance to COM (cm)	14.69 ± 3.75	-

CT = contact time, IC = initial contact, MaxStepFreq = maximum step length × maximum step frequency, **min_accel_magnitude = minimum acceleration magnitude**, Peakforce_CT = peak force **÷** contact time, SD = standard deviation, TAV = thigh angular velocity, VLR = vertical loading rate. - denotes that no data was calculated for this variable across the distance.

## Data Availability

The datasets used and/or analysed during the current study are available from the corresponding author on reasonable request.

## Supplementary Materials

The following supporting information can be downloaded at: https://www.mdpi.com/article/10.3390/sports14080346/s1, Table S1: Regression model for 60 metre sprint time (n = 28). Table S2: Regression model for 60 metre MaxV (n = 28). Table S3: Regression model for 20 metre sprint time (n = 28). Table S4: Regression model for 20 metre maximal velocity (MaxV_20m) (n = 28).

## Abbreviations

CT	contact time
IMU	inertial measurement units
MaxV	maximal velocity
MaxV_20m	maximal velocity across 20 m
MaxStepFreq	maximum step length x maximum step frequency
min_accel_magnitude	minimum acceleration magnitude
peakforce_CT	peak force ÷ contact time
percent_maxV60	percentage of maximal velocity at 20 m relative to 60 m
TAV	thigh angular velocity
IC	initial contact
SEE	standard error of the estimate
VLR	vertical loading rate
