# Supplementary material for: Emerging Portable Technology Provides Preliminary Predictive Models of Maximum Velocity Sprinting in National Australian Track Athletes: A Foundation for Measuring Sprint Performance in the Field
Source: Sports (Basel). 2026 Aug 11;14(8):346. doi: 10.3390/sports14080346 (PMC13517709; doi:10.3390/sports14080346)
Supplement: Supplementary file 1 [file sports-14-00346-s001.zip › sports-4438067-supplementary.pdf]

Table S1. Regression model for 60 metre sprint time (n = 28).

Predictor	B	SE	β	t	p	95% CI	VIF
Maximal velocity (m/s)	-0.622	0.031	-0.97	-20.15	< 0.001	[-0.686, -0.559]	1.00

$F(1, 26) = 406.17$, < 0.001; $R^2 = 0.940$, adjusted $R^2 = 0.938$, SEE = 0.107; leave-one-out cross-validated $R^2 = 0.932$, optimism = 0.008. B, unstandardized coefficient; β, standardized coefficient; CI, confidence interval; VIF, variance inflation factor.

Table S2. Regression model for 60 metre MaxV (n = 28).

Predictor	B	SE	β	t	p	95% CI	VIF
Normalised peak force (N/kg)	-0.015	2.91×10^{-3}	-0.55	-5.16	< 0.001	[-0.021, -9.00×10^{-3}]	1.66
Vertical loading rate (N/s)	1.71×10^{-5}	2.41×10^{-6}	1.24	7.12	< 0.001	[1.21×10^{-5}, 2.21×10^{-5}]	4.48
Time to peak force (ms)	0.046	0.014	0.52	3.17	0.004	[0.016, 0.076]	3.94

Contact time (ms)	-0.027	0.017	-0.26	-1.61	0.121	[-0.062, 7.72×10^{-3}]	3.87
Thigh angular velocity (rad/s)	0.261	0.111	0.20	2.36	0.028	[0.031, 0.490]	1.08

$F(5, 22) = 25.00$, < 0.001; $R^2 = 0.850$, adjusted $R^2 = 0.816$, SEE = 0.285; leave-one-out cross-validated $R^2 = 0.775$, optimism = 0.075. B, unstandardized coefficient; β, standardized coefficient; CI, confidence interval; VIF, variance inflation factor.

Table S3. Regression model for 20 metre sprint time (n = 28).

**Predictor**	**B**	**SE**	**β**	**t**	**p**	**95% CI**	**VIF**
20 m maximal velocity (m/s)	-0.307	0.034	-0.86	-9.03	< 0.001	[-0.377, -0.237]	1.13
Peak force (N)	-3.13×10^{-5}	4.03×10^{-5}	-0.07	-0.78	0.444	[-1.14×10^{-4}, 5.17×10^{-5}]	1.13

$F(2, 25) = 49.07$, < 0.001; $R^2 = 0.797$, adjusted $R^2 = 0.781$, SEE = 0.078; leave-one-out cross-validated $R^2 = 0.758$, optimism = 0.039. B, unstandardized coefficient; β, standardized coefficient; CI, confidence interval; VIF, variance inflation factor.

Table S4. Regression model for 20 metre maximal velocity (MaxV_20m) (n = 28).

**Predictor**	**B**	**SE**	**β**	**t**	**p**	**95% CI**	**VIF**
Vertical loading rate (N/s)	1.04×10^{-5}	2.88×10^{-6}	0.65	3.63	0.001	$[4.51 \times 10^{-6}, 1.64 \times 10^{-5}]$	1.85
Impulse (kN·s)	0.806	0.882	0.16	0.91	0.370	[-1.014, 2.627]	1.83
Step frequency (Hz)	-0.235	0.176	-0.18	-1.33	0.195	[-0.599, 0.129]	1.08

$F(3, 24) = 11.38$, < 0.001; $R^2 = 0.587$, adjusted $R^2 = 0.536$, SEE = 0.319; leave-one-out cross-validated $R^2 = 0.496$, optimism = 0.091. B, unstandardized coefficient; β, standardized coefficient; CI, confidence interval; VIF, variance inflation factor.
